# Learning Organizational Culture in Greek Public Hospitals

**DOI:** 10.3390/ijerph18041867

**Published:** 2021-02-14

**Authors:** Aspasia Goula, Maria-Aggeliki Stamouli, Dimitra Latsou, Vasiliki Gkioka, Niki Kyriakidou

**Affiliations:** 1 Department of Business Administration, School of Administrative, Economics and Social Sciences, University of West Attica, 12243 Athens, Greece; mstamouli@yahoo.com (M.-A.S.); demilatsou@yahoo.gr (D.L.); vgkioka@gmail.com (V.G.); 2 Leeds Business School, City Campus, Leeds Beckett University, Leeds LS1 3HE, UK; N.Kyriakidou@leedsbeckett.ac.uk

**Keywords:** organizational learning, public hospitals, learning culture, health, patient safety

## Abstract

(1) Background: A learning organizational culture is crucial to the safety of patients and the quality of public health care. The aim of this study was to assess the learning organizational culture and capacity of Greek public hospitals. (2) Materials and Methods: A cross-sectional analysis was carried out in six public general hospitals and stratified sampling was used as the sampling technique. A total of 480 questionnaires were distributed to health care professionals and 380 valid questionnaires were returned (78% response rate). The comprehensive form of the Dimensions of Learning Organization Questionnaire (DLOQ), which was adapted and translated into Greek, was used for data collection in this survey. (3) Results: The level of learning organizational culture and capacity in the health units are very low. All seven dimensions of the learning organizational instrument are lower than the theoretically neutral median (3.0). Health care employees believe that the hospital’s existing culture and management practices do not foster and contribute to continuing learning, which is the fundamental aspect of self-development, department development and performance improvement. (4) Conclusions: Greek public hospitals need to adopt different types of leadership practices and culture in order to be able to facilitate organizational learning. Organizational learning (OL) is based on collaborative working, a culture that encompasses learning as participation in the organizational work practice. This transformation of culture should take place at all levels of learning to enhance results.

## 1. Introduction

Learning Organization is organization that has the ability to use those techniques and processes necessary for turning knowledge into skill. By turning knowledge into a skill an organization can modify itself [1]. When an organization is able to modify itself, it means that it can reshape the production process whenever a change occurs [2]. According to this approach, learning comes through the constant change of thought, perception and behavior that arises as an indirect or direct result of knowledge and experience. Learning means changing [3] and organizational learning (OL) can only be achieved through individual change because the individual has the ability to act after thinking. [4,5].

The first reference concerning the "learning organization" approach was made in 1990 by Peter Senge [6] in his book “The Fifth Discipline” where he describes these organizations as places “where people are constantly expanding their ability to create the results they really want, cultivating new and expansive patterns of thinking where collective ambition is unleashed and people are constantly learning how to coexist”. The need for a Learning Organizational approach arose as conventional thinking at that time was not able to offer longer-term solutions to contemporary problems. The learning organization provides a different perspective on meeting contemporary challenges [7].

However, there is an ongoing debate about the levels of analysis of organizational learning. Many argue that organizational learning is the set of knowledge that individuals acquire within the organization while others believe that it is the knowledge that each individual acquires individually [8]. Hannah and Lester [9] argue that organizational learning is an inherently complex process because it involves connecting diverse and often conflicting individuals, groups, functions, policies, and processes.

### Hospital as a Learning Organization

The implementation of the learning approach in health care organizations aims to improve the level of clinical practice, productivity, lifelong learning and patient safety while at the same time reducing costs through the adoption of a balanced learning organizational process [10]. There are currently many issues that hospitals face in Europe, including the aging population, the growth of chronic diseases and the advancement of health care options through modern technology. The ongoing economic downturn has impacted upon resource capability and increased demand for health services.

Healthcare organizations involve multidisciplinary healthcare professionals forming various interconnected care groups striving to provide safe and reliable treatment [11]. Health care groups need to collaborate and interact with the members of their team and other health care providers in order to perform high-risk coordination processes concerning patient’s health care [12]. When unexpected changes occur, i.e., patient-related or sudden demands for public health crises such as pandemics, they have to be able to alter their operations. Health care organizations must preserve continuity in compliance with operational protocols and evaluate protocols to implement new information based on knowledge development and technology advances.

The quality of patients’ care and safety depends upon the individual health care system in which care is provided. Historically, the unequal and wasteful distribution of financial, human and material resources has impacted negatively upon the Greek healthcare system. Within this context and the prevalent priorities of reducing government expenditure across the health sector (in inpatient and outpatient treatment as well as pharmaceuticals), long-term leadership engagement would be needed to consistently resolve inefficiencies [13]. 

The implementation of a learning organizational process is not feasible if the "learning organization" does not function under a “guiding” vision [14]. A commonly accepted vision and shared values must be accompanied by an effort to achieve a balance between individual and collective needs, enhancing coherence.

The purpose of this study is to assess the potential implementation of the learning organizational approach, as it is defined by Watkins and Marsick [15,16], in public general hospitals. This is to identify the potential implementation barriers and facilitate leadership achieving critical strategic changes to promote patient safety. Therefore, the research questions that arise, about Greek hospitals, are the following:

- a. What is the level of organizational learning culture of Greek public hospitals?
- b. Are there managerial practices and mechanisms that facilitate organizational learning dimensions?
- c. Do demographic characteristics affect organizational learning dimensions?

## 2. Materials and Methods

### 2.1. Participants and Procedure

The survey was carried out in 6 public general hospitals in the region of Attica, Greece. For these hospitals, the evaluation criteria were the wide range of healthcare services they offered and the vast number of patients they routinely treated and accommodated. According to the Greek Ministry of Health [17], in the region of Attica, there are 23 General Public hospitals which, in 2019, provided health care services to 634,691 patients. The 6 hospitals that we selected for our research, provided, the same year, health care services to 275,145 patients (44% of the whole access population). The specific hospitals cover secondary and tertiary as well as specialized primary health needs. The research design was cross-sectional and stratified sampling was used as a sampling technique. Consequently, the population was divided into subgroups (doctors, nurses, administrative and paramedical staff). For each stratum, a simple random sampling was implemented to choose a random sample of each subgroup. The stratification procedure was based on the specialization of the participants. The goal of the approach selected was to improve the sample accuracy by reducing the sampling error. In the above-mentioned hospitals, questionnaires were provided to 487 healthcare professionals. A total of 380 valid questionnaires were returned (this corresponds to a response rate of 78 percent). The research was conducted from 17 December 2019 to 10 March 2020.

All participants provided written informed consent forms through a statement as a part of the survey questionnaire itself before proceeding to complete the survey. Data collection guaranteed anonymity and confidentiality. All subjects had been informed of their rights to refuse or discontinue participation in the study, according to the ethical standards of the Helsinki Declaration.

### 2.2. Research Instrument

The research instrument used in this study for data collection was the systematic Dimensions of Learning Organization Questionnaire (DLOQ) form, which consists of 43 questions that make up the seven organizational learning subscales. These subscales are the following: (i) continuous learning (CL—7 items); (ii) inquiry and dialogue (I&D—6 items); (iii) team learning (TL—6 items); (iv) embedded system (ED—6 items); (v) empowerment (Em—6 items); (vi) system connection (SC—6 items); (vii) strategic leadership (SL—6 items). On 5-point Likert scales, which range from 1—totally disagree to 5—totally agree, the objects included in each dimension were measured [18].

In many different sectors of economic activity and in various countries has the DLOQ been used, tested and validated. Regarding the use of the tool in health care services, research in different countries and cultures has recorded its reliability and validity too. These studies have been able to check the applicability of the DLOQ in culturally diverse cultures by providing the internal consistency of the reliability of each item (the alpha coefficient ranges from 0.71 to 0.91) [12].

In this study, the questionnaire was adapted and translated into Greek in a four-stepped process: forward translation, evaluation, backward translation, and final assessment. Two groups of healthcare experts conducted pilot testing and some changes were made based on their comments. The final version of the DLOQ was shared with the sample under analysis, followed by an examination of its validity and reliability. Two types of validity were evaluated: (1) face validity and (2) validity of constructs. Three leading experts of the health sector examined the face validity of the translated DLOQ and stated that it is characterized by high face validity. Regarding construct validity of the DOLQ, with the use of the Multitrait–Multimethod Matrix, it was proved that all the variables of the same factors are statistically significantly correlated (*p* < 0.001), and their correlation coefficients have moderate to high power ranging between 0.563 and 0.798. Moreover, the discriminant validity was demonstrated as certain correlation coefficients between variables of different factors were found to be higher than that of certain correlation coefficients between variables of the same factors. The internal consistency among the items of the DOLQ range between 0.842 and 0.977 and they are considered to be good to excellent. The findings suggested that, because it is characterized by ample convergent validity and moderate discriminant validity, it can be used for evaluating the learning culture in the Greek health field [12].

### 2.3. Statistical Analysis

Data analysis was carried out with SPSS 26 (IBM, Athens, Greece). The seven dimensions of Organizational Learning were calculated as mean values of the variables/questions that compose each one of them, according to the instructions of the DLOQ questionnaire [18]. Kolomogorov–Smirnov and Shapiro–Wilk tests were used in order to check normality of the distributions of the dimensions of OL and of the distribution of “Years of Experience” variable. These tests showed statistically significant deviation from normality for all of the above variables so the relevant statistical tests were non-parametric (Table 1).

The non-parametric Wilcoxon signed rank test was used, for both ordinal and continuous variables (due to deviation from normality), in order to determine statistically significant differences between the sample and the theoretically neutral median (neutral median = 3.5) and the Mann–Whitney U test was used to determine possible statistically significant differences of the OL dimensions between two independent groups. Finally, the non-parametric Kruskal–Wallis-H test was used to determine whether there are statistically significant differences of the OL dimensions between more than two groups (with post-hoc analysis based on the non-parametric Mann–Whitney U test with Holm–Bonferroni correction).

For the evaluation of possible linear correlation between “Years of Experience” and the dimensions of OL, the bootstrapping correlation method based on the observations (B = 1000 bootstrap samples) was considered as the most appropriate, since the distributions of the variables showed deviation from normality. The same method (bootstrapping regression based on the observations, B = 1000 bootstrap samples) was used to evaluate the possible impact of the “Years of Experience” on the OL dimensions (when linearity is valid), unless all the parametric assumptions for the model of linear regression were met (independence, homoscedasticity and normality of the residuals). In this case a simple linear regression model was calculated. The level of statistical significance was set to α = 0.05.

## 3. Results

### 3.1. Descriptive Analysis of the Sample

The DLOQ was distributed to 487 health professionals in 6 Greek Public Hospitals in the region of Attica and 380 valid questionnaires were returned. Regarding the sampling frame, 70.5% of the respondents were females and 29.5% males. In terms of job title, the majority of the participants (76.3%) were employees, 15.0% were heads of offices and the remaining 7.6% were heads of departments and directors. As anticipated, most of the health professionals in the research (40.0%) were nurses, 31.8% were administrative staff, 21.6% were doctors and the remaining 6.6% were paramedical personnel. It is worth noting that more than half of the participants were university graduates holding a postgraduate title (a M.Sc. and/or a Ph.D.). Concerning age distribution, most of them (43.4%) belonged to the age group of (45–54) and 31.3% to the age group of (35–44). Finally, their average professional experience was 17.16 ± 9.48, with a median value of 16.0 years (Table 2).

Statistical analysis using the one-sample Wilcoxon signed rank test for employees’ opinions regarding the OL level of the units in which they work, showed statistically significant lower values for all the seven dimensions of the OL from the theoretically neutral median (neutral value = 3.0). Although the dimension with the higher mean and median value is ID, it was still lower than the theoretical neutral (Table 3). The values of the dimensions, as displayed in Table 3, showed that the level of OL in the health units was not satisfactory.

### 3.2. Analysis of the Individual Characteristics of OL That Constitute Its Dimensions

The seven questions which represent the “Continuous Learning” dimension are presented in Table 4 that follows. According to this table, it became apparent that all seven questions had a statistically significant lower value from the theoretically neutral median with the exception of: (a) in the hospital, people identified skills they needed for future work tasks and (b) in the hospital people helped each other learn, that had statistically significant higher value.

The dimension “Inquiry and Dialogue” is composed of six questions that are illustrated in Table 5. Statistical analysis using the one sample Wilcoxon signed rank test showed that all questions had a statistically significant lower value from the theoretically neutral median except for: (a) in the hospital people give open and honest feedback to each other and (b) in the hospital people treat each other with respect, for which the test was not statistically significant.

Regarding statistical analysis for the remaining dimensions: (a) Team Learning, (b) Embedded Systems, (c) Empowerment, (d) System connection and (e) Strategic Leadership, where each one is composed of six separate questions (Table 6), implementing the one-sample Wilcoxon sign rank test showed a statistically significant lower value (*p* < 0.05) for all questions from the theoretically neutral median.

### 3.3. The Influence of Demographic Factors on the Organizational Learning Dimensions

The Mann–Whitney U test was used to evaluate the impact of “gender” on the subscales of Organizational Learning. The test was not statistically significant for any of the subscales, (U_CL_ = 13,303.00, *p* = 0.517, U_ID_ = 13,258.00, *p* = 0.732, U_TL_ = 13,577.00, *p* = 0.943, U_ES_ = 13,003.00, *p* = 0.261, U_Em_ = 13,730.00, *p* = 0.936, USC = 13,794.00, *p* = 0.849, U_SL_ = 14,119.00, *p* = 0.617) an outcome which suggests that gender does not affect Organizational Learning. It is worth noting that, women score higher on “Continuous Learning”, “Inquiry and Dialogue”, “Embedded Systems” and “System Connection” while men on “Team Learning”, “Empowerment” and “Strategic Leadership”.

Based on the analysis with Kruskal–Wallis-H, in order to study the effect of “Job Title” factor on the subscales of Organizational Learning, it is proven that “Job Title” does not affect Organizational Learning, since the test it not statistically significant for any of its subscales (H_CL_ = 4320, *p* = 0.229, H_ID_ = 3729, *p* = 0.292, H_TL_ = 3378, *p* = 0.337, H_ES_ = 2923, *p* = 0.404, H_Em_ = 3078, *p* = 0.380, H_SC_ = 1645, *p* = 0.649, H_SL_ = 3518, *p* = 0.318). However, it is worth mentioning that higher values were recorded for the heads of departments in all the dimensions, except for “Inquiry and Dialogue”, in which higher scores were recorded for the heads of offices.

Regarding the “Age Categories” factor, statistical analysis using the Kruskal–Wallis-H test showed statistically significant differences only for the subscales of “Inquiry and Dialog” (H = 9234, *p* = 0.026) and “Team Learning” (H = 8160, *p* = 0.043). The corresponding post-hoc statistical analysis based on non-parametric Mann–Whitney U tests, with the Holm–Bonferroni correction method), for “Inquiry and Dialog” subscale showed a statistically significant difference for the age categories pair (35–44 vs. 55–64) (*p* = 0.0102, α_holm-bonferroni_ = 0.083) with the older age groups recording higher scores. It is worth noting that the post hoc analysis for the pair (45–54 vs. 55–65) was not statistically significant, although this was a marginal result (*p* = 0.012, α_holm-bonferroni_ = 0.01). For this case also the older age groups scored higher.

The same post hoc analysis for “Team Learning” subscale revealed statistically significant differences for the pairs (i) 35–44 vs. 45–54 (*p* = 0.009, α_holm-bonferroni_ = 0.01) and (ii) 45–54 vs. 55–64 (*p* = 0.007, α_holm-bonferroni_ = 0.083), where in both cases the older age groups had higher values.

The “Education Level” factor did not seem to affect Organizational Learning since Kruskal–Wallis-H test was not statistically significant for any of its subscales (H_CL_ = 5767, *p* = 0.217, H_ID_ = 2405, *p* = 0.662, H_TL_ = 4252, *p* = 0.373, H_ES_ = 7338, *p* = 0.119, H_Em_ = 2652, *p* = 0.618, H_SC_ = 2853, *p* = 0.583, H_SL_ = 3179, *p* = 0.528).

Statistical analysis for testing for linear correlation between “Professional Experience” and “Organizational Learning” subscales with 1000 bootstrap samples on the observations, was significant, positive and with very small bias, for all the OL subscales except for the subscales of “Empowerment” and “System Connection” (Table 7). A more detailed study and illustration of these relations is presented in the regression analysis that follows.

As shown in Table 8, standardized residuals were not normally distributed for the subscales of “Team Learning” (Z = 0.57, *p* = 0.010 and W = 0.99, *p* = 0.02), “Embedded Systems” (Z = 0.53, *p* = 0.022 and W = 0.97, *p* < 0.0001) and “Strategic Leadership” (Z = 0.68, *p* = 0.001 and W = 0.97, *p* < 0.001). Thus, for these subscales, as already mentioned, bootstrap regression models (B = 1000 samples on the observations) were calculated. Moreover, since the assumption of normality was not violated nor any of the other linear regression assumptions for the subscales of “Continuous Learning” (Z = 0.35, *p* = 0.02 and W = 0.99, *p* = 0.205) and “Inquiry and Dialogue” (Z = 0.31, *p* = 0.02 and W = 0.99, *p* = 0.22), a simple linear regression model can be carried out for these subscales.

### 3.4. Simple Linear Regression Models

A simple linear regression was calculated in order to predict “Continuous Learning” based on “Professional Experience”, (b = 0.179, t (343) = 23,758). A significant regression equation was suggested (F (1, 343) = 11,332, *p* < 0.001), with an R-Square = 0.032. The predicted value of “Continuous Learning” was 2.585 + 0.019 *(Professional Experience) (Table 9) where experience was measured in years, which means that Continuous Learning increased by 0.019 units for each year of experience. Only 3.2% of the variation in “Continuous Learning” was explained by which means that the effect size of the experience on “Continuous Learning” was very low [19,20].

Regarding the “Inquiry and Dialogue” subscale, a simple linear regression equation was conducted based on “Professional Experience” (b = 0.123, t (340) = 26,278). A significant regression equation was determined (F (1, 340) = 5,192, p = 0.023), with an R-Square = 0.015. The predicted value of “Inquiry and Dialogue” was 3.066 + 0.014 *(Professional Experience) (Table 9) where experience was measured in years, which means that “Inquiry and Dialogue” increased by 0,014 units for each year of experience. Professional Experience did not explain a significant amount of variance in “Inquiry and Dialogue” (R-Square = 1.5%), meaning that the effect size of the Experience on Continuous Learning was very low. 

### 3.5. Bootstrap Regression Models

A bootstrap regression analysis was conducted to predict “Team Learning” based on “Professional Experience”, since the assumption of normality was violated. A significant regression equation was calculated (F (1, 340) = 8.270, *p* = 0.004), with an R-Square = 0.024. The value of correlation coefficient was very low ((R = 0.154, Bias Corrected and Accelerated (BCa) 95% CI (0.045, 0.250)) (Table 10) implying a low level of prediction accuracy. The predicted value of “Team Learning” was 2.620 + 0.017 * (Professional Experience) (Table 10) where experience was measured in years, which means that “Team Learning” increased by 0.017 units for each year of experience. Only 2.4% of the variation in “Team Learning” was explained by “Professional Experience”, meaning that the effect size of the experience on “Team Learning” was low. Moreover, bias to the bootstrap correlations was very low (Table 10).

In order to predict “Embedded Systems” based on “Professional Experience, a bootstrap regression model was calculated. A significant regression equation was suggested (F(1, 340) = 6.128, *p* = 0.014) with an R-Square = 0,018 (Table 10). The value of correlation coefficient was significant but low (R = 0.133, BCa 95% CI (0.019, 0.236)) (Table 10), implying a low level of prediction accuracy. The predicted value of “Embedded Systems” was 2.441 + 0.016 *(Professional Experience) (Table 10), where experience was measured in years, which means that “Embedded Systems” increased by 0.016 units for each year of experience. “Professional Experience” did not explain a significant amount of variance in “Embedded Systems”, (R-Square = 1.8%), a result which means that the effect size of the “Professional Experience” on “Embedded Systems” was low. Bias to the bootstrap correlations was also very low (Table 10).

Finally, a bootstrap regression model was also estimated to predict “Strategic leadership” based on “Professional Experience”. A significant regression equation was calculated (F(1, 350) = 4.853, *p* = 0.028), with an R-Square = 0.014 (Table 10). The value of the correlation coefficient was significant but small (R = 0.117, BCa 95% CI (0.004, 0.216)) (Table 10), which means that the accuracy of the prediction was low. The predicted value of “Strategic Leadership” was 2.465 + 0.016 *(Professional Experience) (Table 10), where experience was measured in years, which means that “Strategic Leadership” increased by 0.016 units for each year of “Professional Experience”. Only 1,4% of the variation in “Strategic Leadership “ was explained by “Professional Experience” (Table 10), which means that the effect size of the “Professional Experience” on “Strategic Leadership” was low. From Table 10 it is apparent that bias to the bootstrap correlations was very low.

## 4. Discussion

The key components for the healthcare organizations to be in line with the current circumstances and requirements of globalization are the immediate response to change, innovation, user–patient orientation, quality improvement, the ability to adapt to the new conditions and, specifically, organizational learning of new business data [21]. The ability to learn is crucial because, due to the continuous development in science and medicine, the existing expertise and skills can easily become outdated in this area [22]. In addition, organizational learning has been strongly recommended by the Institute of Medicine as a promising tool for improving health systems and delivering better results for patients [23].

In this study, 380 health employees from 6 general hospitals in the region of Attica participated, aiming to identify the organizations’ ability to learn as it is defined by Watkins and Marsick [15,16]. The research tool used was the extensive form of the DLOQ (Dimensions of Learning Organization Questionnaire), which consists of 43 questions that compose the seven subscales of Organizational Learning [18]. 

Overall, it was made clear that the level of the organizational learning culture in the health units under study was very low, since all the seven organizational learning dimensions had lower median values than the theoretically neutral median (median = 3.0).

Specifically, the “continuous learning” subscale had a mean score of 2.92, (median = 2.86), a value which was lower compared to the findings of other relevant studies [24,25,26,27,28]. This result indicated that Greek public hospitals do not encourage continuing education and learning programs for health professionals [18], despite the fact that “continuous learning” is the fundamental factor for improving the capability of a healthcare organization to achieve employees’ satisfaction, to respond promptly to changes and thus to enhance its productivity and its efficiency [1,15,29,30]. 

Although the dimension “inquiry and dialogue”, had the highest mean value (mean = 3.1, median = 3.33) among all the organizational learning subscales, this value was still lower than the theoretically neutral median. This finding was in line with the studies of Leufven et al. study [26] and Watkins et al [24] but disagreed with findings of other studies [25,27,28]. The low value of this dimension indicated that research opportunities in Greek public hospitals are not at a satisfactory level, while at the same time the exchange of knowledge among employees is not encouraged either. Additionally, the fact that the 55–64 age group had the highest score suggests that older employees tend to share their feelings and thoughts more than younger ones. Moreover, they give their colleagues the opportunity to openly express their views and opinions and they encourage research and foster innovation within the health unit they work in [18]. Accordingly, in Alas R. and Vadi M.’s [31] survey, it has been shown that older employees, in terms of organizational learning, make for a better group of learners than younger ones.

The score of the "team learning" dimension was substantially lower (mean = 2.92, median = 2.83) than the corresponding findings in other studies [24,25,26,27,28]. The above results gave an indication that team learning within the Greek health units was not at a satisfactory level; as such, it should be further encouraged because team-level learning is key to achieving organizational-level learning since the skills, the experience, and achievements accomplished by a continuously learning team can then be shared throughout the organization, thus establishing a learning norm.

However, the culture of Greek public hospitals is plagued by its internal structure, compliance with laws and procedures, emphasis on the control system and also predictability and consistency, which limits or totally excludes employees’ involvement in decision making. All of the above exert a significant drag on the transformation of health care units to learning organizations [32]. The learning organization has a supportive organizational culture, which promotes learning, continuously, dynamically and collectively [33]. Greek healthcare employees find it difficult to understand the value of teamwork, disregard its necessity and act individually. It has become also apparent that the permanent status of employment had a negative effect on team learning as it perpetuated a shortage of ideas and therefore limited knowledge and vision. With a lack of vision, “collectivity” and “teamwork” were concepts that cease to exist. At this point it is also worth noting that, in the Greek public sector, dissemination of team learning is negligible as there are no systems to allow it [22].

The “embedded system” dimension, had a mean value of 2.74 (median = 2.50) which was much lower than the corresponding results of other relevant surveys. [24,25,26,27,28]. This was a finding which indicated that the mechanisms for measuring and exchanging learning were missing [18]. For a public organization, like the Greek hospitals, with entrenched bureaucracy culture in its structure, it is difficult to be reformed into a flexible and rapidly evolving learning organization. The quality of learning organizational depends on organizational culture, which facilitates or inhibits learning [34]. It is proven that cultures that oppose change have impeded new working models, inventions and new technologies. [35,36]. The creation, dissemination and utilization of learning demand a “friendly” culture and therefore a culture of participation is needed where the organization’s systemic approach prevails.

As regards the "empowerment" subscale, it was shown that it had the lowest mean score of all the organizational learning subscales (mean = 2.49, median = 2.33), which was also lower than the findings of other surveys [24,25,26,27,28]. Empowerment ensures that employees were involved in creating, owning and implementing a common vision and also that were motivated by leaders to learn, understand and assimilate the tasks and duties for which they were responsible [18]. In order to achieve this, Greek public hospitals require a leadership pattern that will strengthen the collaborations between individuals and will ensure that the vision of the hospital is common and understood by all [37].

Regarding the “system connection” subscale, statistical analysis showed that it had a mean score of 2.70 (median = 2.80) which was lower than the corresponding results of similar studies [24,25,26,27,28]. This finding indicated that Greek public hospitals are disconnected from their environment and do not use evidence to change their working practices [18]. They are far from the holistic integrative perspective proposed by Watkins and Marsick [15,16], where in order to facilitate continuous learning and change, a learning organization has the capacity to incorporate individuals and systems [1]. Therefore, Greek public hospitals should emphasize the conditions prevailing in the internal and external environment of the organization, its culture, and the development of programs for fundamental organizational changes in order to succeed as learning organizations [38]. 

Lastly, the "strategic leadership" dimension had a mean value of 2.75 (median = 2.67), which was also lower than the results of other studies [24,25,26,27,28]. This outcome implied that leaders either have not been able to provide strategic leadership for learning or have not been able to create that kind of climate and culture within the organization which facilitates organizational learning. [18]. Transactional leadership is the dominant form in most Greek public hospitals that does not facilitate learning and a number of them have been resistant to transformational efforts [39], and yet according to Bass, only transformative organizations are primed, competent and eager to adapt [40]. Therefore, in order to enhance organizational learning, mostly hospitals, should concentrate on transformational leadership. A catalytic agent and a mentor is a transformational leader within the learning organization [41], who fosters dialog and communication among the members of the organization [42] and encourages an appropriate environment for innovative teams [43].

As regards the impact of “gender”, “education level” and “job title”, it has been shown that they did not influence the dimensions of organizational learning. Professional experience, though, was found to have a significant impact on all the organizational learning dimensions, except for the subscales of “Empowerment” and “System Connection”, but with a weaker effect. These results were in contrast to the research of Watkins et al [24]. In Greek public hospitals, employees with more experience tend to have more favorable views on continuous learning, dialogue, team learning and strategic aspects of leadership.

### Limitations of Study

There are some limitations in this analysis that need to be discussed. The survey findings apply to six Attica general hospitals, so the results can therefore only be restricted to these hospitals and may not reflect the culture of learning organization of all public hospitals in the region. In addition, further studies should examine how organizational learning ideals can be effectively applied to other fundamental concepts such as job satisfaction or organizational commitment.

## 5. Conclusions

This study made an attempt to evaluate the learning organizational culture in Greek public health care units. The findings suggested that Greek public hospitals, according to the theoretical framework of the Dimensions of Learning Organization Questionnaire, need to adopt different models of leadership practices and different models of culture in order to be able to facilitate organizational learning. Organizational learning is based on collaborations, teamwork, accountability and the culture of participation. This transformation of culture must take place at all levels of learning; that is, at the individual level, at the group level, at the organizational level and finally at the working environment level, in order to facilitate an effective learning process with tangible results.

## Figures and Tables

**Table 1:** Normality tests for organizational learning (OL) dimensions and for Years of Experience.

OL Dimensions	Kolmogorov–Smirnov ^a^			Shapiro–Wilk		
	Statistic	df	Sig.	Statistic	df	Sig.
CL	0.060	364	0.003	0.987	364	0.003
I&D	0.057	362	0.006	0.989	362	0.008
TL	0.075	362	0.000	0.983	362	0.000
ES	0.085	364	0.000	0.963	364	0.000
Em	0.101	365	0.000	0.939	365	0.000
SC	0.095	364	0.000	0.958	364	0.000
SL	0.086	374	0.000	0.956	374	0.000
Years of Experiece	0.082	358	0.000	0.969	358	0.000

^a^ Lilliefors Significance Correction. CL: Continuous Learning, I&D: Inquiry and Dialogue, TL: Team Learning, ES: Embedded System, Em: Empowerment, SC: System Connection, SL: Strategic Leadership.

**Table 2:** Sampling frame description.

Socio-Demographic Characterists		Frequency	Percent
**Gender**	Male	112	29.5
	Female	268	70.5
**Position level**	Employee	290	76.3
	Head of office	57	15.0
	Head of Department	14	3.7
	Director	15	3.9
	Missing Values	4	1.1
**Specialty**	Doctor	82	21.6
	Nurse	152	40.0
	Administrative staff	121	31.8
	Paramedical staff	25	6.6
**Education level**	Secondary education	83	21.8
	Technological education	91	23.9
	Higher education (university degree)	68	17.9
	M.Sc.	91	23.9
	Ph.D.	32	8.4
	Missing Values	15	3.9
**Age group**	25–34	46	12.1
	35–44	119	31.3
	45–54	165	43.4
	55–64	46	12.1
	Missing Values	4	1.1

**Table 3:** One-sample Wilcoxon signed rank OL dimensions.

OL Dimensions	Mean	Median	*p*
CL	2.92	2.86	<0.001
ID	3.31	3.33	<0.001
TL	2.92	2.83	<0.001
ES	2.74	2.50	<0.001
Em	2.49	2.33	<0.001
SC	2.70	2.50	<0.001
SL	2.75	2.67	<0.001

CL: Continuous Learning, I&D: Inquiry and Dialogue, TL: Team Learning, ES: Embedded System, Em: Empowerment, SC: System Connection, SL: Strategic Leadership.

**Table 4:** Comparison of the individual characteristics of “Continuous Learning” dimension to the neutral value with the use of the one-sample Wilcoxon sign rank test.

	Questions	Mean	Mdn	W *	*p*
**Continuous Learning**	In the hospital people openly discuss mistakes in order to learn from them	3.06	3.00	24,419.50	<0.001
	In the hospital, people identify skills they need for future work tasks	3.34	4.00	31,689.00	0.048
	In the hospital people help each other learn	3.76	4.00	43,950.00	<0.001
	In the hospital people can get money and other resources to support their learning	2.23	2.00	8086.50	<0.001
	In the hospital, people are given time to support learning	2.76	3.00	17,079.50	<0.001
	In the hospital people view problems in their work as an opportunity to learn	2.98	3.00	21,775.50	<0.001
	In the hospital people are rewarded for learning	2.39	2.00	9739.00	<0.001

* Wilcoxon sign rank test.

**Table 5:** Comparison of the individual characteristics of the “Inquiry and Dialogue” dimension to the neutral value with the use of the one-sample Wilcoxon sign rank test.

	Questions	Mean	Mdn	W *	*p*
**Inquiry and Dialogue**	In the hospital people give open and honest feedback to each other.	3.43	4.00	34,953.00	0.552
	In the hospital people listen to others’ views before speaking.	3.24	3.00	28,428.50	<0.001
	In the hospital people are encouraged to ask “why” regardless of rank.	3.17	3.00	27,380.00	<0.001
	In the hospital whenever people state their view, they also ask what others think.	3.30	3.00	30,354.00	0.011
	In the hospital people treat each other with respect.	3.30	4.00	34,517.00	0.994
	In the hospital people spend time building trust with each other.	3.47	3.00	29,510.00	0.004

* Wilcoxon sign rank test.

**Table 6:** Comparison of the individual characteristics of: (a) Team Learning, (b) Embedded Systems, (c) Empowerment, (d) System connection and (e) Strategic Leadership dimensions with the use of the one-sample Wilcoxon sign rank test.

**Team Learning—Characteristics**	**Mean**	**Mdn**	W *	***p***
In the hospital teams/groups have the freedom to adapt their goals as needed	3.19	3.00	26,939.00	<0.001
In the hospital teams/groups treat members as equals, regardless of rank, culture, or other differences	2.90	3.00	20,301.50	<0.001
In the hospital teams/groups focus both on the group’s task and on how well the group is working	3.20	3.00	26,991.00	<0.001
In the hospital teams/groups revise their thinking as a result of group discussions or information collected	3.33	3.00	31,376.00	0.04
In the hospital teams/groups are rewarded for their achievements as a team/group	2.51	2.00	11,463.00	<0.001
In the hospital teams/groups are confident that the organization will act on their recommendations	2.53	2.00	10,962.50	<0.001
**Embedded Systems—Characteristics**	**Mean**	**Mdn**	W *	***p***
Το use two-way communication on a regular basis	3.05	3.00	23,702.00	<0.001
The hospital enables people to get needed information at any time quickly and easily	2.89	3.00	18,864.50	<0.001
The hospital maintains an up-to-date database of employee skills	2.63	2.00	14,667.00	<0.001
The hospital creates systems to measure gaps between current and expected performance.	2.38	2.00	9,538.50	<0.001
The hospital makes its lessons learned available to all employees	3.10	3.00	25,666.00	<0.001
The hospital measures the results of the time and resources spent on training	2.55	2.00	12,385.00	<0.001
**Empowerment—Characteristics**	**Mean**	**Mdn**	W *	***p***
The hospital recognizes people for taking initiative	2.73	3.00	16,198.00	<0.001
The hospital gives people choices in their work assignments	2.64	2.00	14,486.00	<0.001
The hospital invites people to contribute to the organization’s vision	2.53	2.00	12,622.50	<0.001
The hospital gives people control over the resources they need to accomplish their work	2.33	2.00	8,216.00	<0.001
The hospital supports employees who take calculated risks	2.37	2.00	9,390.00	<0.001
The hospital builds alignment of visions across different levels and work groups	2.38	2.00	9,178.50	<0.001
**System Connection—Characteristics**	**Mean**	**Mdn**	W *	***p***
The hospital helps employees balance work and family	2.84	3.00	19,613.50	<0.001
The hospital encourages people to think from a global perspective	2.51	2.00	11,492.00	<0.001
The hospital encourages everyone to bring the customers’ views into the decision-making process	2.75	3.00	15,477.00	<0.001
The hospital considers the impact of decisions on employee morale	2.65	2.00	14,170.50	<0.001
The hospital works together with the outside community to meet mutual needs	2.94	3.00	20,869.00	<0.001
The hospital encourages people to get answers from across the organization when solving problems	2.59	2.00	13,392.00	<0.001
**Strategic Management—Characteristics**	**Mean**	**Mdn**	W *	***p***
The hospital leaders generally support requests for learning opportunities and training	3.01	3.00	23,262.50	<0.001
The hospital leaders share up-to-date information with employees about competitors, industry trends, and organizational directions.	2.57	2.00	12,719.50	<0.001
In the hospital leaders empower others to help carry out the organization’s vision	2.71	3.00	15,778.00	<0.001
In the hospital leaders mentor and coach those they lead	2.54	2.00	13,143.50	<0.001
In the hospital leaders continually look for opportunities to learn	2.74	2.00	17,909.00	<0.001
Leaders ensure that the organization’s actions are consistent with its values	2.97	3.00	22,435.50	<0.001

* Wilcoxon sign rank test.

**Table 7:** The impact of Professional Experience on Organizational Learning subscales (Bootstrap 1000 samples).

Bootstrap Correlations	CL	ID	TL	ES	Em	SC	SL
Correlation Coefficient	0.179	0.123	0.154	0.133	0.04	0.081	0.117
BCa * 95% CI **	(0.075, 0.281)	(0.003, 0.244)	(0.045, 0.250)	(0.019, 0.236)	(−0.068, 0.155)	(−0.032, 0.199)	(0.004, 0.216)
Bias	0.001	0.001	−0.001	−0.0002	−0.001	0.002	−0.003

* Bias Corrected and Accelerated, ** Confidence Interval.

**Table 8:** Standardized residuals normality tests.

Tests of Normality							
Line	Standardized Residuals	Kolmogorov–Smirnov			Shapiro–Wilk		
		Statistic	df	Sig.	Statistic	df	Sig.
1	ZRE_CL	0.035	345	0.200	0.994	345	0.205
2	ZRE_ID	0.031	342	0.200	0.993	342	0.120
3	ZRE_TL	0.057	342	0.010	0.990	342	0.020
4	ZRE_ES	0.053	342	0.022	0.973	342	0.000

**Table 9:** Summary statistics of simple linear regression coefficients.

Subscale		B	95% CI	b	t	*p*	Tolerance	VIF
**Continuous Learning**	(Constant)	2.585	(2.371, 2.799)		23.758	0.000		
	Professional Experience	0.019	(0.008, 0.029)	0.179	3.366	0.001	1.00	1.00
R^2^ = 3.2%, R^2^ Adjusted = 0.029, CI = Confidence Interval for B, Durbin-Watson = 1.905								
**Inquiry and Dialogue**	(Constant)	3.066	(2.836, 3.295)		26.278	0.000	1.00	1.00
	Professional Experience	0.014	(0.002, 0.025)	0.123	2.279	0.023		
R^2^ = 1.5%, R^2^ Adjusted = 0.012 CI = Confidence Interval for B, Durbin–Watson = 1.790								

**Table 10:** Summary Statistics of regression coefficients for bootstrap regression based on observations (B = 1000).

Bootstrap for Coefficients							
Model		B	Bootstrap				
			Bias	Std. Error	Sig. (2-Tailed)	BCa * 95% Confidence Interval	
						Lower	Upper
**Team Learning**	(Constant)	2.620	0.003	0.109	0.001	2.414	2.846
	Professional Experience	0.017	0.000	0.006	0.004	0.006	0.028
R^2^ = 1.5%, R^2^ Adjusted = 0.021, Durbin–Watson = 1.814 * Bias Corrected and Accelerated							
**Embedded Systems**	(Constant)	2.441	0.000	0.114	0.001	2.211	2.670
	Professional Experience	0.016	0.000	0.007	0.019	0.002	0.029
R^2^ = 1.3%, R^2^ Adjusted = 0.015, Durbin–Watson = 1.849							
**Strategic Leadership**	(Constant)	2.465	0.007	0.131	0.001	2.206	2.748
	Professional Experience	0.016	0.000	0.008	0.044	0.001	0.029
R^2^ = 1.1%, R^2^ Adjusted = 0.014, Durbin–Watson = 1.769							

* Bias Corrected and Accelerated.

## Data Availability

Restrictions apply to the availability of these data. Data were collected from Post-Graduate Program “Health and Social Care Management” of the Department of Business Administration of the University of West Attica and are available from Goula A. with the permission of MSc Program.

## References

[1] Yang B., Watkins K., Marsick V. (2004). The construct of the learning organization: Dimensions measurement and validation. Hum. Resour. Dev. Q..

[2] Schein E. (1993). On Dialogue, Culture, and Organizational Learning. Organ. Dyn..

[3] Noe R., Hollenbeck J., Gerhart B. (2014). Human Resources Management.

[4] Quinn R. (1996). Deep Change.

[5] Jones G. (2010). Organizational Theory, Design, and Change.

[6] Senge P.M. (2006). The Fifth Discipline: The Art and Practice of the Learning Organization.

[7] Bui H., Baruch Y. (2010). Creating learning organizations: A systems perspective. Learn. Organ..

[8] Li S., Easterby-Smith M., Bartunek J. (2009). Research methods for organizational learning: The transatlantic gap. Manag. Learn..

[9] Hannah S., Lester P. (2009). A multilevel approach to building and learning organizations. Leadersh. Q..

[10] Wijayanti A., Sara R., Handayani A.P., Windy W.T., Sulaiman M., Wulandari R.D. (2020). Learning organization to maintain full accreditation of public health center. Eurasian J. Biosci..

[11] Ratnapalan S., Uleryk E. (2014). Organizational learning in health care organizations. Systems.

[12] Goula A., Stamouli M.A., Latsou D., Gkioka V., Sarris M. (2020). Validation of Dimensions of Learning Organization Questionnaire (DLOQ) in health care setting in Greece. J. Public Health Res..

[13] Economou C., Kaitelidou D., Karanikolos M., Maresso A. (2017). Greece: Health System Review. Health Systems in Transition.

[14] Weick K., Quinn R. (1999). Organizational change and development. Annu. Rev. Psychol..

[15] Watkins K.E., Marsick V.J. (1993). Sculpting the Learning Organization: Lessons in the Art and Science of Systemic Change.

[16] Watkins K.E., Marsick V.J. (1996). Action: Creating the Learning Organization.

[17] Ministry of Health (2019). Hospitalized Beds—Days of Hospitalization. https://www.moh.gov.gr/articles/bihealth/stoixeia-noshleytikhs-kinhshs.

[18] Marsick V.J., Watkins K.E. (2003). Demonstrating the value of an organization’s learning culture. Adv. Dev. Hum. Resour..

[19] Moore D., Notz W., Flinger A. (2013). The Basic Practice of Statistics.

[20] Zikmund W. (2000). Business Research Methods.

[21] Goula A. (2014). Health Care Services’ Organizational Culture.

[22] Vassalou L. (2001). The learning organization in health-care services: Theory and practice. J. Eur. Ind. Train..

[23] Lyman B., Hammond E.L., Cox J.R. (2019). Organizational learning in hospitals: A concept analysis. J. Nurs. Manag..

[24] Watkins K., Milton J., Kurz D. (2009). Diagnosing the learning culture in public health agencies. Int. J. Contin. Lifelong Learn..

[25] Kumar J.K., Basavaraj P., Gupta R., Singla A. (2016). An insight into health care setup in national capital region of India using Dimensions of Learning Organizations Questionnaire (DLOQ)—A cross sectional study. J. Clin. Diagn. Res..

[26] Leufvén M., Vitrakoti R., Bergstrom A., Ashish K.C., Malquist M. (2015). Dimensions of learning organizations questionnaire (DLOQ) in a low-resource health care setting in Nepal. Health Res. Policy Syst..

[27] Goula A., Katelouzou D., Pierrakos G., Latsou D., Dimakou S., Stamouli M.A., Natsis C. (2019). Analysis of the relationship between transformational leadership and learning organization in health care services. Strategic Innovative Marketing and Tourism.

[28] Song J.H., Joo B.K., Chermack T.J. (2009). The dimensions of learning organization questionnaire (DLOQ): A validation study in South Korean context. Hum. Resour. Dev. Q..

[29] Camps J., Luna-Arocas R. (2012). A Matter of Learning: How Human Resources Affect Organizational Performance. Br. J. Manag..

[30] Kontoghiorghes C., Awbrey S.M., Feurig P.L. (2005). Examining the relationship between learning organization characteristics and change adaptation, innovation, and organizational performance. Hum. Resour. Dev. Q..

[31] Alas R., Vadi M. (2006). The impact of organizational culture on organizational learning and attitudes concerning change from an institutional perspective. Int. J. Strateg. Chang. Manag. IJSCM.

[32] Goula A. (2007). Hospital Administration & Management—The Greek Experience & Practice.

[33] Marquardt M.J. (1996). Building the Learning Organization: A Systems Approach to Quantum Improvement and Global Success.

[34] Lipshitz R., Popper M. (2000). Organizational learning in a hospital. J. Appl. Behav. Sci..

[35] Holmberg L. (1997). Health-Care Processes: A Study of Medical Problem-Solving in the Swedish Health-Care Organization.

[36] Benders J., Van Hootegem G. (1999). Teams and their context: Moving the team discussion beyond existing dichotomies. J. Manag. Stud..

[37] Chermack T.J., Lynham S.A., Van der Merwe L. (2006). Exploring the relationship between scenario planning and perceptions of learning organization characteristics. Futures.

[38] Watkins K.E., Kim K. (2018). Current status and promising directions for research on the learning organization. Hum. Resour. Dev. Q..

[39] Schwartz R., Trumblin T. (2002). The power of servant leadership to transform health care organizations for the 21st-century economy. Arch. Surg..

[40] Bass B.M. (2000). The future of leadership in learning organization. J. Leadersh. Stud..

[41] Senge P., Roberts C., Ross R.B., Smith B.J., Kleiner A. (1994). The Fifth Discipline Field Book: Strategies and Tools for Building a Learning Organization.

[42] Megheirkouni M. (2017). Leadership styles and organizational learning in UK for-profit and non-profit sports organizations. Int. J. Organ. Anal..

[43] Tushman M.L., Nadler D.A. (1986). Organizing for innovation. Calif. Manag. Rev..

